# Crystallographic Anisotropy Dependence of Interfacial Sliding Phenomenon in a Cu(16)/Nb(16) ARB (Accumulated Rolling Bonding) Nanolaminate

**DOI:** 10.3390/nano12030308

**Published:** 2022-01-18

**Authors:** Rahul Sahay, Arief S. Budiman, Izzat Aziz, Etienne Navarro, Stéphanie Escoubas, Thomas W. Cornelius, Fergyanto E. Gunawan, Christian Harito, Pooi See Lee, Olivier Thomas, Nagarajan Raghavan

**Affiliations:** 1 Xtreme Materials Lab, Engineering Product Development, Singapore University of Technology and Design (SUTD), Singapore 487372, Singapore; 2 Industrial Engineering Department, BINUS Graduate Program, Bina Nusantara University, Jakarta 11480, Indonesia; fegunawan@binus.edu (F.E.G.); charito@binus.edu (C.H.); 3 Department of Manufacturing and Mechanical Engineering and Technology, Oregon Institute of Technology, Klamath Falls, OR 97601, USA; 4 School of Materials Science and Engineering, Nanyang Technological University, Singapore 308232, Singapore; izzat@ntu.edu.sg (I.A.); pslee@ntu.edu.sg (P.S.L.); 5 Aix-Marseille University, Université de Toulon, CNRS, IM2NP, 13013 Marseille, France; etienne.navarro@gmail.com (E.N.); stephanie.escoubas@im2np.fr (S.E.); thomas.cornelius@im2np.fr (T.W.C.); olivier.thomas@univ-amu.fr (O.T.); 6 nano-Micro Reliability Lab (MRL), Engineering Product Development, Singapore University of Technology and Design (SUTD), Singapore 487372, Singapore; nagarajan@sutd.edu.sg

**Keywords:** nanoindentation, finite element analysis, nanolayers, pile-up, plastic deformation, interfacial sliding

## Abstract

Nanolaminates are extensively studied due to their unique properties, such as impact resistance, high fracture toughness, high strength, and resistance to radiation damage. Varieties of nanolaminates are being fabricated to achieve high strength and fracture toughness. In this study, one such nanolaminate fabricated through accumulative roll bonding (Cu(16)/Nb(16) ARB nanolaminate, where 16 nm is the layer thickness) was used as a test material. Cu(16)/Nb(16) ARB nanolaminate exhibits crystallographic anisotropy due to the existence of distinct interfaces along the rolling direction (RD) and the transverse direction (TD). Nanoindentation was executed using a Berkovich tip, with the main axis oriented either along TD or RD of the Cu(16)/Nb(16) ARB nanolaminate. Subsequently, height profiles were obtained along the main axis of the Berkovich indent for both TD and RD using scanning probe microscopy (SPM), which was later used to estimate the pile-up along the RD and TD. The RD exhibited more pile-up than the TD due to the anisotropy of the Cu(16)/Nb(16) ARB interface and the material plasticity along the TD and RD. An axisymmetric 2D finite element analysis (FEA) was also performed to compare/validate nanoindentation data, such as load vs. displacement curves and pile-up. The FEA simulated load vs. displacement curves matched relatively well with the experimentally generated load–displacement curves, while qualitative agreement was found between the simulated pile-up data and the experimentally obtained pile-up data. The authors believe that pile-up characterization during indentation is of great importance to documenting anisotropy in nanolaminates.

## 1. Introduction

Nanolaminates are studied both theoretically and experimentally on the virtue of their unique properties such as high strength [1,2], high fracture toughness [3,4,5], extreme deformability [3,6,7], shock resistance [8], electrical conductivity [9], high temperature strength retention [10], high-temperature creep [11] and high resistance to radiation damage [12]. These nanolaminates have been used for radiation damage protection [13], and potential applications include hybrid diffusive–displacive helium outgassing [14] and structural materials [10,13]. These nanolaminates typically do not follow the rule of mixtures, i.e., the volume fraction of each component does not determine their properties, but rather their individual layer thickness and interfacial structure [15,16]. Typically, layer thickness can drastically alter the mechanical properties of nanolaminates [17,18]. For example, in the layer thickness range of 100 to 10 nm, the hardness and strength increase with the reduction in the layer thickness. In contrast, for layer thicknesses below ~10 nm, the hardness and strength strongly depend on the mechanisms responsible for the motion of dislocations in the layers. Furthermore, as the layer thickness decreases, the interfacial density multiplies, which affects both the mechanical and physical behavior of the nanolaminate [19]. Three different interfacial structures are usually formed in nanolaminates: incoherent, semi-coherent and coherent interfaces, which depend on the lattice parameters, crystallographic structure and the possible slip system between adjacent layers. The coherent interface with similar crystallographic structures and lattice parameters commonly found in FCC/FCC nanolaminates (e.g., Cu/Ni [20]) allows dislocations to accumulate and move across the interface. On the other hand, an incoherent interface comprises lattice structures with a high degree of lattice mismatch, which could lead to discontinuity among the slip systems at the interface and could hinder the movement of dislocations across the interface. Examples of incoherent interfaces include FCC/BCC Cu(16)/Nb(16) ARB nanolaminate [21,22].

Various techniques have been used to prepare such nanolaminates. These include atomic layer deposition [23,24], physical vapor deposition [25,26], accumulative roll bonding [27], electrodeposition [28,29], and chemical vapor deposition [30]. Further, various methods using severe plastic deformation (SPD) are employed for the production of ultrafine grained/nano grained nanolaminates with superior mechanical properties. These SPD-based methodologies include equal channel angular pressing (ECAP) [31], high-pressure torsion (HPT) [32] and accumulative roll bonding (ARB) [33,34,35,36]. Of these methodologies, ARB is especially significant, as it can generate continuous sheets of ultrafine grained/nano grained bimetallic nanolaminate that can be scaled up to generate large billets of nanolaminate for a wide range of industrial applications. Accumulative roll bonding (ARB) uses a top-down process where millimeter-scale metal sheets are repeatedly stacked and rolled down to nanometric length scales. This fabrication method is used for the fabrication of bimetallic nanolaminates such as Mg/Al [37], Al/Cu [38], Ti/Al [39], Cu/Ag [40], Cu/Nb ARB [27], and Cu/Ni [41]. During ARB, the metal sheets undergo severe plastic deformation in the rolling direction (RD) compared to the transverse direction (TD) [33,34,35,36] generating an anisotropic behavior. The material tested in this study was an accumulative roll bonded Copper(Cu)/Niobium(Nb) nanolaminate having a 16 nm final layer thickness for both Cu and Nb. Cu(16)/Nb(16) ARB nanolaminates with an incoherent interface are studied computationally and experimentally on virtue of their excellent strength, fracture toughness, radiation resistance, and thermal stability [3,12,42].

Initially, the mechanical properties of multilayer nanolaminates were extensively studied by indentation [43,44]. Later, these indentation measurements were used to develop various dislocation-dependent models for multilayer nanolaminates to fit experimentally obtained flow strength. Nevertheless, the inhomogeneous stresses [45,46,47] generated during indentation can complicate the study of the anisotropy of multilayered nanolaminates. To solve this problem, nanoindentation is performed with the main axis of the indentation aligned along either the TD or RD of the anisotropic nanolaminates to study their pile-up and then to relate it to the dissimilarity of plasticity noted along the TD and RD. It is very likely (in the case of ARB nanolaminates) that the indentation axis aligned along the rolling and transverse directions results in a highly anisotropic deformation [48,49,50,51], which could lead to plastic deformation-generated impressions such as sink-in or pile-up, characteristic to Cu(16)/Nb(16) ARB nanolaminates.

Here, the anisotropic nature of Cu(16)/Nb(16) ARB nanolaminates was investigated both experimentally and by finite element analysis. The anisotropic behavior of incoherent Cu(16)/Nb(16) ARB nanolaminates is of great interest, as remarkable differences in properties can be achieved along the rolling (RD) and transverse directions (TD) [33]. In this study, besides nanoindentation experiments, we also performed an axisymmetric 2D finite element analysis to compare the experimental results (such as load-displacement curves and pile-up) with the simulation data. It was found that the simulated load vs. displacement curves showed quantitative agreement with the indentation tests for both TD and RD with respect to indentation depth. The trend of pile-up from the simulation was correctly reproduced for both RD and TD, which was then related to the variation of plasticity along these directions. Moreover, the Oliver–Pharr scheme was applied to determine the hardness and reduced elastic modulus of the Cu(16)/Nb(16) ARB nanolaminate.

## 2. Materials and Methods

### 2.1. Materials

**Figure 1:**
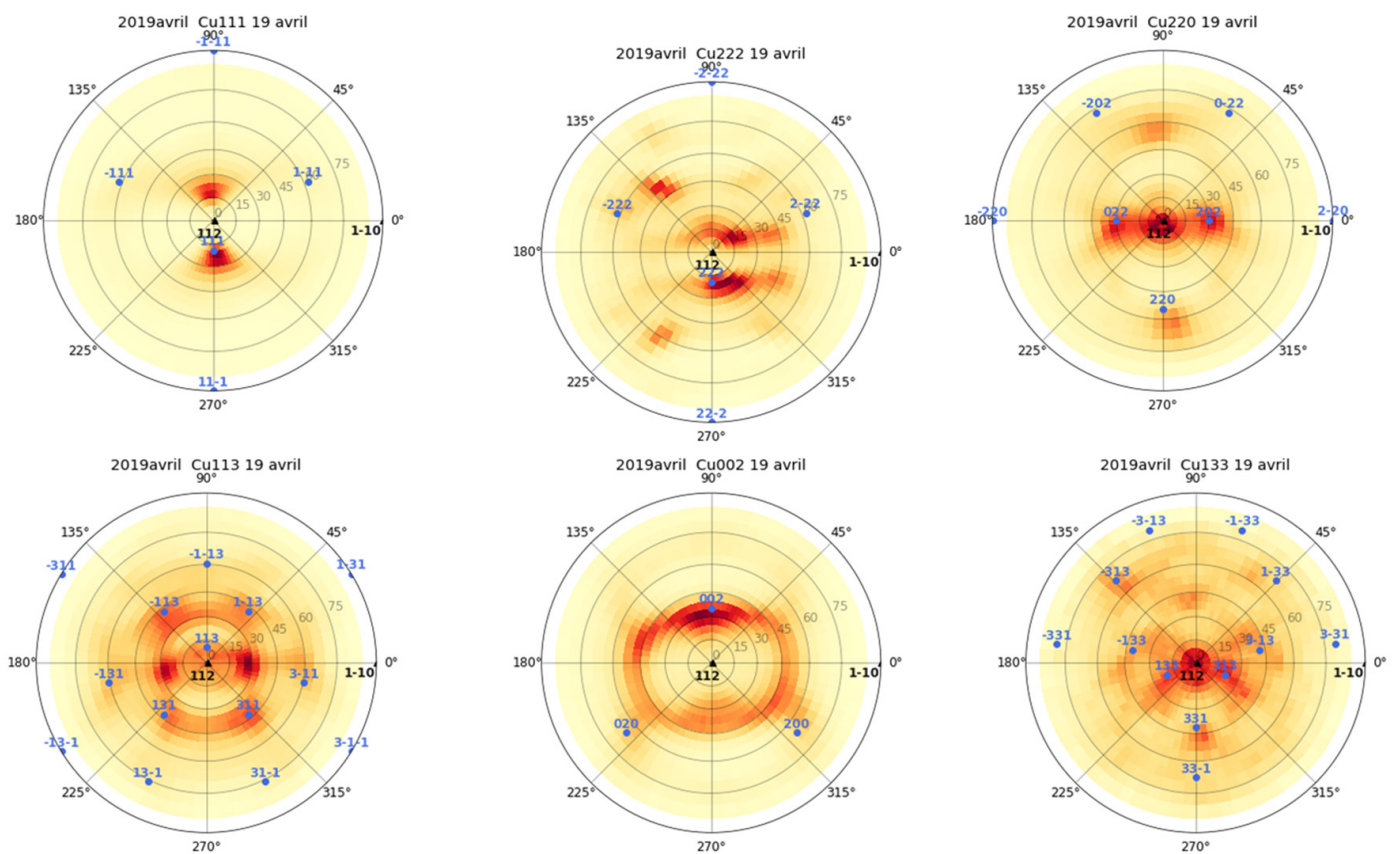
Pole figure measurements of copper in Cu(16)/Nb(16) ARB nanolaminate using a 4-axis diffractometer [57].

**Figure 2:**
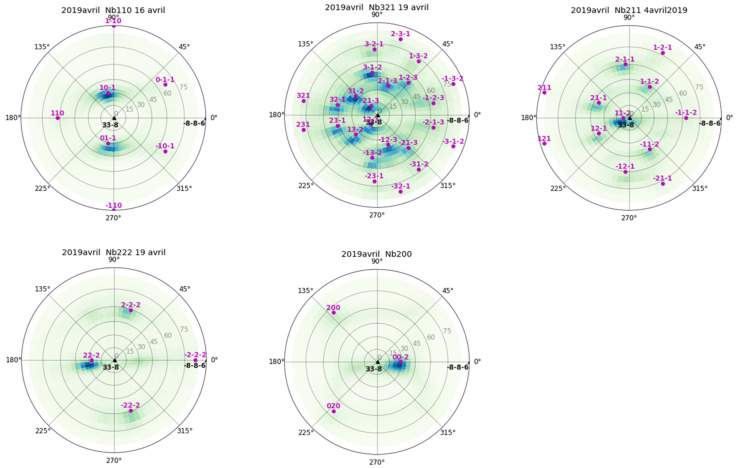
Pole figure measurements of niobium in Cu(16)/Nb(16) ARB nanolaminate using a 4-axis diffractometer [57].

Cu(16)/Nb(16) ARB nanolaminates were prepared at the Los Alamos National Laboratory, USA. Cu(16)/Nb(16) ARB nanolaminates were produced by rolling a 1 mm pure Nb metal sheet inserted between two 0.5 mm pure Cu metal sheets. These rolled samples were repeatedly halved and restacked and then re-rolled (with the same rolling direction) to obtain Cu(16)/Nb(16) ARB nanolaminates [21]. The ARB sample used in the analysis was Cu(16)/Nb(16) ARB nanolaminate with a 16 nm nominal layer thickness. A 4-axis diffractometer (X’Pert, PanAlytical, Malvern, UK) (Supplementary Data, Figure S1) equipped with a Cu anode (λ = 1.54184 Å) and operated at 40 kV and 30 mA was used for the pole figure measurements, as shown in Figure 1 and Figure 2 for copper and niobium, respectively. Here, the angle psi was varied from 0° to 75°, while phi was varied from 0° to 360° (phi = 0° corresponds to TD and phi = 90° corresponds to RD) to obtain the diffraction patterns of the predominant crystallographic planes. Cu(16)/Nb(16) interfaces consist of a combination of {112} <111> Cu || {112} <110> Nb and {552} <110> Cu || {112} <110> Nb [42,52]. Typically, in the ARB process, the grains are found to elongate extensively along the RD compared to TD, which was confirmed by grain aspect ratios exceeding 1:50:5 (ND:RD:TD) at layer thicknesses ≤65 nm [53]. This observed anisotropy in both texture and grain morphology arises from the plane strain deformation that occurs during rolling [54,55]. Typically, for Cu(16)/Nb(16) ARB samples, the stress required to shear the layers along the RD is theoretically infinite, compared to 1.2 GPa along TD [56].

### 2.2. Nanoindentation

In nanoindentation, a rigid indenter penetrates a Cu(16)/Nb(16) ARB nanolaminate. The nanoindentation load and displacement are recorded uninterruptedly throughout the loading and unloading cycle. The obtained load vs. displacement curve is then analyzed through the Oliver–Pharr scheme to acquire its hardness and reduced Young’s modulus of the sample as a function of indentation depth. These indentation tests were executed using a Hysitron Triboindenter (TI 950, Bruker Inc., Billerica, MA, USA) with a Berkovich tip (tip diameter = 100 nm) in continuous stiffness measurement mode. Initially, calibration was performed using a standard quartz sample to obtain tip area function for the Berkovich tip and the Triboindenter compliance to calculate the contact stiffness by means of the Oliver–Pharr scheme. Then, the indentations were executed under displacement control at a loading rate and an unloading rate of 5 and 10 nm/s, respectively, with displacement and load resolutions of ~0.2 and ~30 nN, respectively. The distance between two indentations was ~20 μm to elude contact amongst their individual plastic zones generated during the indentation. Furthermore, the ratio of nanolaminate height to maximum indentation depth was greater than ~10 to avoid any substrate effect on the attained experimental data.

### 2.3. Surface Pile-Up Profile

**Figure 3:**
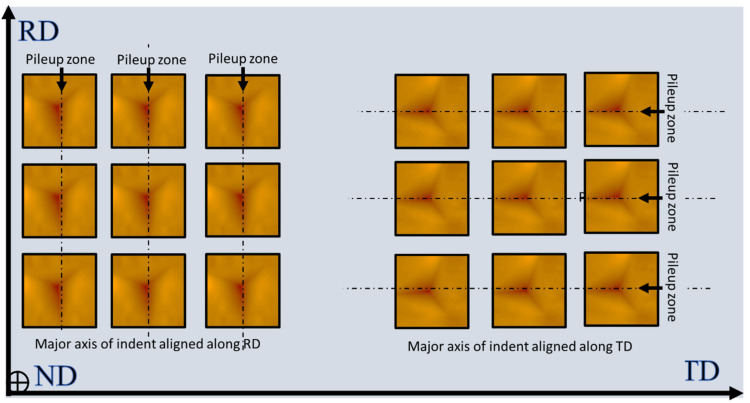
Schematic showing the nanoindentation performed along the RD and TD of a Cu(16)/Nb(16) ARB nanolaminate by aligning the major axis of the Berkovich tip along the TD and RD obtained using SPM. The height profiles were then plotted along the major axis of the indentations (represented as dashed lines) for RD and TD as shown in Section 3.3, and later used to measure the pile-up along these directions.

A scanning probe microscope (SPM) contained within the Triboindenter was then employed to record the three-dimensional profiles of the indentations. SPM was performed in contact mode, with a nominal contact force of 2 μN. An area of 3 × 3 μm^2^ including one indentation was scanned to acquire profiles of indentation with high accuracy. Focused ion beam (FIB) polishing was used to polish the surface of the Cu(16)/Nb(16) ARB nanolaminate before the indentations. During FIB, the sample surface was cleaned using a low current (20 pA, 30 kV). Indentation profiles were then obtained on the polished Cu(16)/Nb(16) ARB nanolaminate surface by aligning the major axis of the Berkovich tip along either the TD or the RD of the Cu(16)/Nb(16) ARB nanolaminate (see Figure 3). These 3D profiles of the indentations obtained through SPM were then used to create height profiles along the major axis of the indents for both the RD and TD, as depicted by dashed line in Figure 3. The representative height profiles along the TD and RD are plotted in Figure shown in Section 3.3. These height profiles were then used to measure the pile-up along the RD and TD. Next, the experimental pile-up data for Cu(16)/Nb(16) ARB nanolaminate were compared to the simulation results.

### 2.4. TEM + EDX Sample Preparation and Analysis

The samples after indentation were then used to make TEM samples along the TD and RD with a focused ion beam (FIB, FEI Nova NanoLab 600i DualBeam, Hillboro, OR, USA). The TEM samples were sliced along the major axis of the indentation along RD and TD (shown with a dashed line in Figure 3). For the Berkovich indentation, a thin layer of protective Pt was first applied to the sliced TEM samples. Then, a long rectangular shape (~10 × ~5 µm) was cut from the sliced TEM samples, extending from one corner to the end of the indentation along the major axis. Two trenches were cut on both sides of this rectangular pattern. The material formed between the trenches was then thinned to 1 μm. The sample was then transferred to Cu lift-out grids. It was then thinned to a final thickness of 100 nm (at 30 kV with 48 pA), which was then examined using a TEM (JEOL 2100F, Tokyo, Japan with integrated Oxford EDX, Abingdon, UK) at 200 kV in STEM mode.

### 2.5. Simulation of Nanoindentation of Cu(16)/Nb(16) ARB Nanolaminate

**Figure 4:**
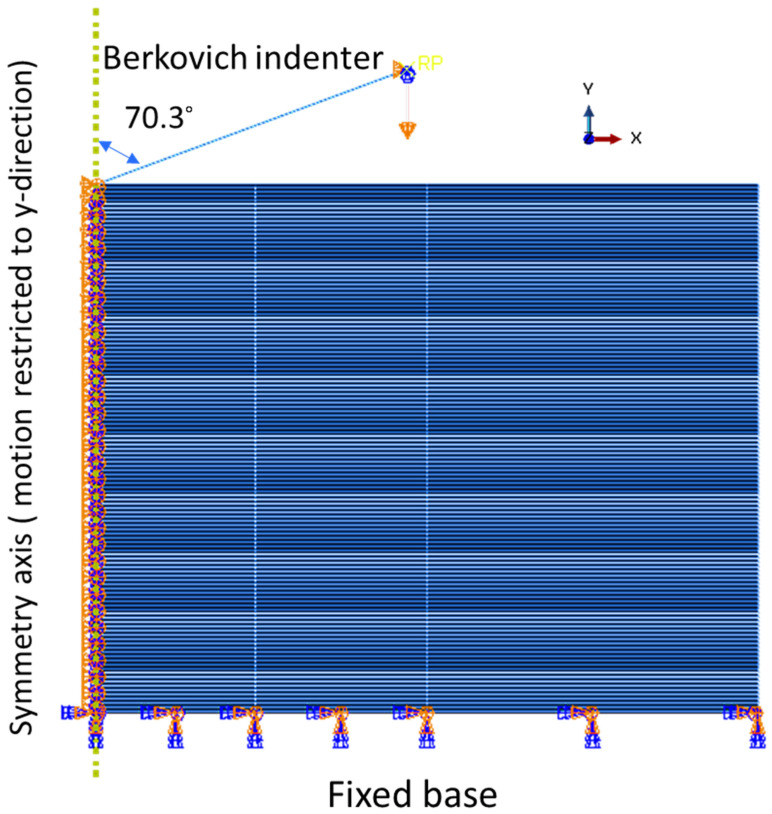
An illustration of sample geometry and loading configuration for the Cu(16)/Nb(16) ARB nanolaminate indentation simulation.

Nanoindentation can mainly deliver statistics about the overall behavior of nanolaminates. Hence, finite element modeling is used to correlate the inhomogeneous stress state obtained during indentation with the mechanical properties of the layer (Cu and Nb) and properties of the Cu(16)/Nb(16) ARB interface. Figure 4 displays an illustration of the sample geometry with loading arrangement for 2D axisymmetric simulation of nanoindentation. The model encompassed 121 alternating layers of Cu and Nb. The layer thickness was the same for both materials: ~16 nm. For the mechanical properties of layers and interface, we used the data published in the previous work of our group by Radchenko et al. [58]. Nevertheless, in the instance of TD, due to higher yield strength, linear plasticity was used in the present analysis, as shown in Table 1. Each individual layer of Cu and Nb was modelled as isotropic and elastoplastic with kinematic hardening. On the other hand, power law plasticity (*n* = 3, *k* = 10,000 for both Cu and Nb) was used for RD since it has higher plasticity (lower yield strength) compared to TD. The properties of the Cu(16)/Nb(16) ARB interface are summarized in Table 2. The Cu(16)/Nb(16) ARB interface was modeled as cohesive surface-to-surface contact [58]. Cohesive surface-to-surface contacts can be demarcated by five characteristic constraints as the maximum normal and tangential (shear) stress, the normal and tangential (shear) stiffness, and the damage degradation rate, which are shown in Table 2. The normal and shear stiffness for the RD and TD beams were kept high to ensure the convergence of the model. The damage degradation rate was adjusted so that the simulated load-displacement curves matched the experimental load-displacement curves. The maximum normal stress did not matter because hard contact criterion of “no separation” was used in the model. The maximum shear for RD was chosen to be sufficiently large to constrain the interfacial shear, while the maximum shear for TD was set to 2.0 GPa to account for the interfacial shear predicted by Demkowicz [56]. Furthermore, the anisotropic behavior of the Cu(16)/Nb(16) ARB nanolaminates was established through molecular static/dynamic simulation by Wang et al. [59]. They noted that the interfacial shear resistance of the Cu(16)/Nb(16) ARB nanolaminate was highly anisotropic in nature. Therefore, the strengthening/hardening mechanism of the incoherent Cu/Nb interface is a function of its anisotropy.

Here, nanoindentation with a Berkovich tip was modeled by an elastoplastic finite element analysis using ABAQUS^®^ CAE 2021 FEA software. Although a 3D model is preferred for nanoindentation simulation, previous work has shown that assuming a conical tip with the same area function as the Berkovich tip yields similar results [60]. Therefore, a quasi-static (2D) axisymmetric finite element model (FE) was utilized, as shown in Figure 4. The sample used was a hypothetical cylinder, equivalent to a rectangle, in an axisymmetric model with a height of 3.9 µm and a radius of 5 µm. The sample size was large enough to avoid the interaction in the elastic-plastic zone generated by the indentation, free surfaces and substrate. The Berkovich tip (tip diameter = 100 nm) was modelled as a rigid cone with a half apex angle of 70.3°, which was estimated as similar ratio of indentation depth to contact area as the Berkovich tip used in the experiments [61]. The tip, with a 100 nm tip diameter, was shortened to a height of 2.5 µm. The friction between indenter and sample was 0.1, a value commonly used for nanoscale contacts [62]. Previous studies have indicated that the friction coefficient does not severely modify hardness/Young’s modulus values [63]. A characteristic mesh for the axisymmetric 2D nanoindentation model consisted of bilinear axisymmetric quadrilaterals with 4 nodes, reduced integration, and hourglass controls (CAX4R). The mesh was intended to grow coarser with distance from the Berkovich tip to enhance the precision of numerical simulations in areas of high plasticity while requiring a comparatively small computation time. The minimum element size was 8 nm × 10 nm below the indenter. The bottom surface of the sample was immovable, and motion along the symmetry axis was constrained to the y direction, y being the normal direction (see Figure 4). The top and right sides of the specimen were free to move, and the movement of the indenter was restricted to the y-direction. The movement of the indenter into the specimen was recorded for each increment of displacement to obtain a load vs. displacement plot analogous to the results of the indentation experiments. Finite element modeling (FE) has proven to be an effective tool to elucidate the stress and damage evolution in applications where in-situ experimental observations are not possible, or when they are only theoretically possible, but in reality, almost impossible, or at least impractical [64,65,66,67,68].

## 3. Results

### 3.1. Nanoindentation Analysis of Cu(16)/Nb(16) ARB Nanolaminate

The reduced Young’s modulus and hardness of the Cu(16)/Nb(16) ARB nanolaminate were measured along the RD and TD using nanoindentation [69]. The factors affecting the reduced Young’s modulus and hardness of the Cu(16)/Nb(16) ARB nanolaminates were the thickness of layers and the structure of the interfaces. The reliance of hardness on the layer thickness can be divided into three main categories, which are commonly observed for both FCC/BCC (Cu/Cr [70], Cu/Nb ARB [17]) and FCC/FCC (Cu/Ag [71], Cu/Ni). The first category is the Hall–Petch relationship (valid from submicron to micrometer, in this case, hardness α *h*^−1/2^, where *h* is the layer thickness), where hardening is associated with the accumulation of dislocations at interfaces in the nanolaminate [72,73]. The second category is called a confined layer slip (CLS), which applies to the length scale of 100 to 10 nm. In the CLS regime, due to small layer thickness, the dislocations are typically permitted to slide in the distinct layers of the multilayer nanolaminates [74], here, hardness α ln(*h*/*b*)/*h* where *b* is the length of the Burgers vector [17]. The third category is effective for few nanometers, where a single dislocation is allowed to cross the inter-layer interface in the nanolaminates [75]. The hardness of the nanolaminate (100 < *h* < 10 nm) belonging to confined layer strengthening/hardening mechanism grows with a reduction in h. Similarly, for Cu/Nb ARB nanolaminate, hardness was found to increase with a reduction in the layer thickness. For instance, Beyerlein et al. [42] associated the hardness of Cu/Nb ARB nanolaminates with layer thickness from 714 nm down to 7 nm. The hardness increased with the decrease in the layer thickness and reached an extremely high value of 5 GPa at a layer thickness of 7 nm. This value is typically 5 to 10 times higher than that of Cu or Nb.

**Figure 5:**
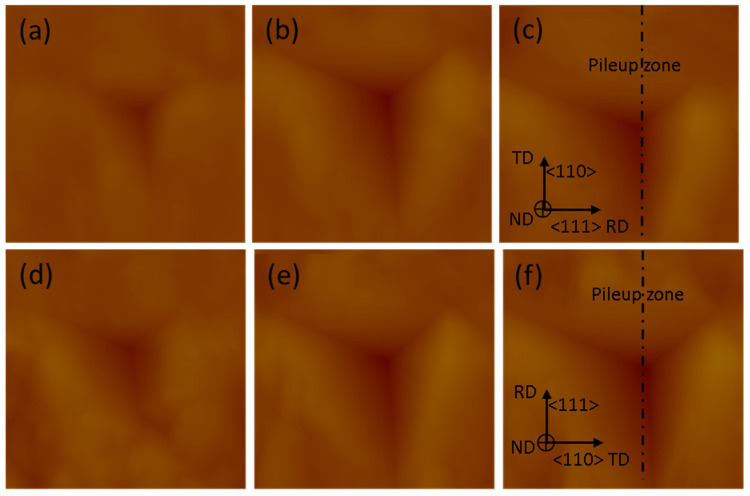
Figure shows scanning probe microscopy (SPM) images of the indentations performed along either the TD or RD of a Cu(16)/Nb(16) ARB nanolaminate. The indention depths were (**a**) 200 nm, (**b**) 300 nm and (**c**) 400 nm for TD and (**d**) 200 nm, (**e**) 300 nm and (**f**) 400 nm for RD. The SPM images with scanned area 3 × 3 μm^2^ were attained after nanoindentation to precisely plot the height profiles along the major axis of indents revealed by the dashed line, and later the corresponding pile-up heights were obtained. Crystallographic directions are specified for Cu.

**Figure 6:**
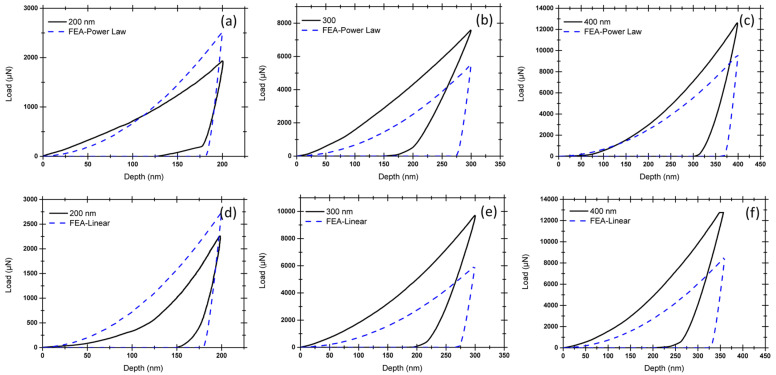
Comparison between the experimental and simulated load vs. displacement curves for the RD and TD of Cu(16)/Nb(16) ARB nanolaminate. The indention depths were (**a**) 200 nm, (**b**) 300 nm and (**c**) 400 nm for RD and (**d**) 200 nm, (**e**) 300 nm and (**f**) 400 nm for TD Cu(16)/Nb(16) ARB nanolaminate.

In the present study, the reduced elastic modulus and hardness of Cu(16)/Nb(16) ARB nanolaminate were determined using nanoindentation. The representative images of the indentations with the main axis of the indenter oriented along either RD or TD with respect to indentation depths (200, 300 and 400 nm) are shown in Figure 5. The corresponding load vs. displacement plots for nanoindentation depths (200, 300 and 400 nm) are depicted in Figure 6. These load vs. displacement curves were employed to estimate the reduced Young’s modulus and hardness by means of the Oliver–Pharr method. The reduced Young’s modulus and hardness of the Cu(16)/Nb(16) ARB nanolaminate for both the RD and TD for the indentation depth (200, 300 and 400 nm) are shown in Figure S2 of the Supplementary Materials. It was found that the hardness and reduced Young’s modulus were in general agreement with the hitherto quantified results [42], although, of course, the previous study [42] did not differentiate between RD and TD in their nanoindentation experiments. In addition, we found that the reduced elastic modulus and hardness along TD were always higher than those of RD for indentation depths of 200, 300 and 400 nm. Furthermore, the average hardness along RD was lower than that along TD, which is reliable considering the lesser yield strength measured along RD as compared to TD [76,77].

### 3.2. Simulation of Indentation on 16 nm Cu(16)/Nb(16) ARB Nanolaminate

**Figure 7:**
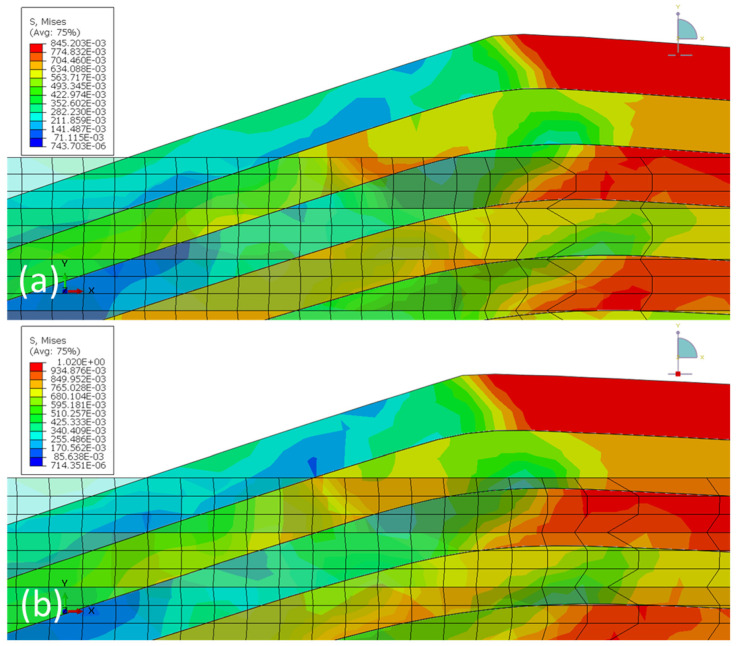
Pile-up distribution along the (**a**) RD and (**b**) TD for an indenter depth of 400 nm. The figure shows initial surface of the sample (with mesh) and the final surface of the sample (only feature edges) superimposed on each other to depict the pile-up. The corresponding videos (Video S1 to Video S6) of the evolution of von Mises’s stress along the TD and RD of the Cu(16)/Nb(16) ARB nanolaminate for all indentation depths are included in the Supplementary Materials. Furthermore, the figures depicting pile-up distribution for nanoindentation depths 200 and 300 nm are also included in the Supplementary Materials. The unit of the data shown in the legend is GPa.

The model was validated by comparing the empirically obtained load vs. displacement plots of nanoindentation with simulated plots of RD and TD with respect to indentation depth (see Figure 6). The simulated load-displacement curves agreed reasonably well with the experimental data for the TD and RD of the Cu(16)/Nb(16) ARB nanolaminates. Nevertheless, the large plastic deformations that occurred during loading were not fully captured by the model. Figure 7 shows a contrast of the von Mises stress along the TD and RD before and after reaching the maximum indentation of 400 nm. The von Mises stress values were slightly higher for TD compared to RD at the same penetration depth (see also Figures S3 and S4 in the Supplementary Materials for the von Mises stress contours before and after indentation for the indentation depth of 200 and 300 nm, respectively). The slightly higher von Mises stress in the case of TD compared to RD can be attributed to its high yield stress. Figure 7 shows that a high pile-up was obtained for RD compared to TD, which can be attributed to the lower yield strength of RD compared to TD [76].

Moreover, when the nanolaminate was subjected to pure compressive load, the softer layer first deformed plastically, while the hard layer remained elastic, so the softer layers determined the mechanical properties of the nanolaminate. Only when the softer layer had sufficiently strain hardening did the harder layers begin to affect the mechanical properties of the nanolaminate. However, during nanoindentation, high plastic deformation occurs at the indentation tip, which affects both the hard and soft layers, creating an inhomogeneous stress state [78]. This proposes that in nanolaminates, layers of dissimilar materials should deform together upon indentation. This inhomogeneous co-deformation may lead to severe thinning of the layers and squeezing of the layers in the radial direction, resulting in the formation of pile-up at the edges of the indents, as was the case with RD. On the other hand, along the TD, homogeneous co-deformation could be hindered by the presence of interfacial slip [79]. However, the presence or absence of co-deformation was not visible in the simulations for either the RD or the TD, which could be attributed to the absence of crystal plasticity in the model.

### 3.3. Pile-Up of Indentations along TD and RD of the Cu(16)/Nb(16) ARB Nanolaminate

**Figure 8:**
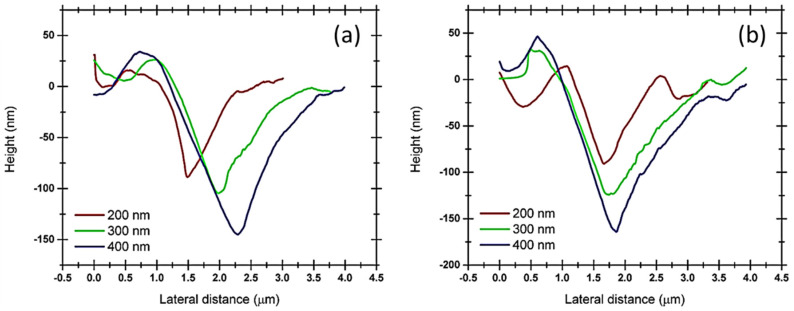
Height profiles obtained through SPM along the major axis of the Berkovich tip for both the RD and TD of Cu(16)/Nb(16) ARB nanolaminate, which were later used for measuring pile-up heights with respect to nanoindentation depth along the (**a**) TD and (**b**) RD of Cu(16)/Nb(16) ARB nanolaminate.

**Figure 9:**
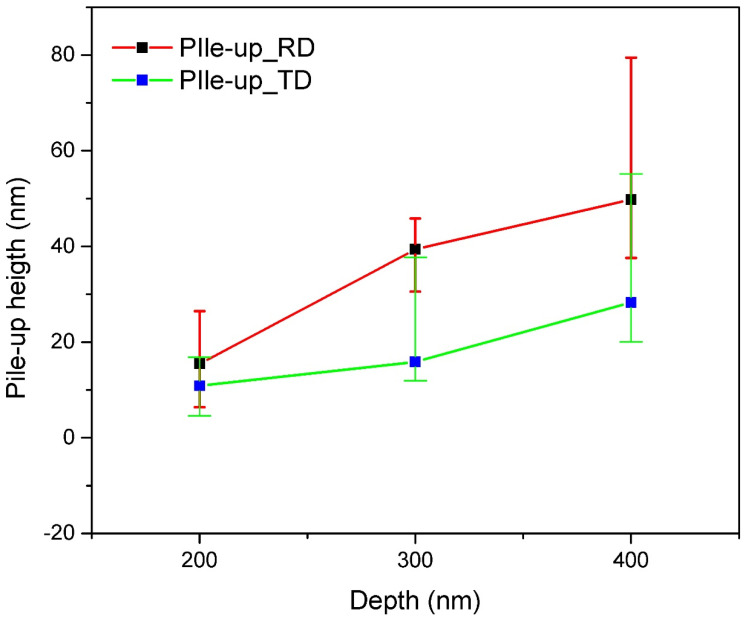
The figure depicts the pile-up heights along the TD and RD of Cu(16)/Nb(16) ARB nanolaminate. Indention depths used for the comparison of pile-up heights are 200, 300, and 400 nm, respectively.

Scanning probe microscopy (SPM) was used to obtain 3D profiles of the indentations along the RD and TD of the Cu(16)/Nb(16) ARB nanolaminate in contact scanning mode. Height profiles were obtained along the major axis of the indentations (see Figure 5). Figure 8 displays the height profile along the RD and TD of Cu(16)/Nb(16) ARB nanolaminate with respect to discrete nanoindentation depths such as 200, 300 and 400 nm. At indentation depths less than 200 nm, particularly at the depths of 50 and 100 nm, the pile-up heights were very low, and in few tests, they were similar to the roughness (surface) of the sample, so they could not be clearly distinguished by SPM. Furthermore, the maximum indentation depth was constrained by the maximum achievable load. In the case of the Cu/Nb nanolaminate (along TD), the maximum achievable load was already reached at the applied indentation depth of 400 nm, as can be seen in Figure 6f. The pile-up heights increased with increasing nanoindentation depth for both the TD and the RD (see Figure 9). A comparison of the pile-up heights shows an increase for RD compared to TD for all indentation depths investigated in this study.

## 4. Discussion

### 4.1. Nanoindentation Performed along TD and RD of Cu(16)/Nb(16) ARB Nanolaminate

Dislocation nucleation and its propagation during the nanoindentation of nanolaminates is highly intricate and not well understood. Typically, harder, and work-hardened materials pile up, while soft and easily hardened materials sink in [80]. During indentation, dislocations are generated in the vicinity of the indenter tip. Later, these dislocations can spread along the available slip planes, which are determined by the load, the corresponding resolved shear stress along the slip plane, and the interactions among these dislocations. When the applied resolved shear stress is higher than the critical resolved shear stress (CRSS), these dislocations may spread along the slip plane and transfer downward in the sample or slide across the surface of an indentation, creating pile-up. For Cu(16)/Nb(16) ARB nanolaminate, when the applied shear stress is higher than CRSS, three scenarios are possible: (a) rotation of the layers to activate the available slip system, which then allows dislocation transfer across the interface, (b) movement of the dislocation along the layers, or (c) deposition of dislocations at the interface between the layers. For a highly work-hardened RD, the ability of dislocations to transfer across the layers or deposit in the interface could be lower than along the TD, which would increase the tendency of the dislocations to slide along the layer. This would induce the co-deformation of layers due to high interfacial shear strength and would accumulate material at the periphery of the indent, leading to higher pile-up along the RD, as shown in Figure 9. On the other hand, less work-hardened and low interfacial shear strength TD could allow dislocations to be deposited in the interface rather than contributing to the plasticity of the material, as is the case with RD, and lower co-deformation due to low interfacial shear strength leads to lower accumulation compared to RD.

To further elaborate the analysis, the increased pile-up for RD in comparison to TD can be attributed to material and interfacial plasticity. The material plasticity of nanolaminates depends on the thickness of an individual layer [17], which has been divided into three regimes. The first is the Hall–Petch regime, where the layer thickness is more than 100 nm, which allows the accumulation of dislocations [70,81,82]. The second regime, where the layer thickness is between 100 nm and 10 nm, is associated with the CLS, where a dislocation glides within the layers [83,84,85]. The last regime occurs below 10 nm layer thickness, where a single dislocation crosses across the interface into an adjacent layer. The CLS model relevant to Cu(16)/Nb(16) ARB nanolaminate drives the applied shear stress (*σ_cls_*) [17] essential to move a glide loop of Burgers vector *b* restricted to a single layer as:

(1) $$\sigma_{cls}=\frac{\mu b}{8\pi h}\left(\frac{4-\vartheta }{1-\vartheta }\right)\left[\ln\frac{\beta h}{b}\right]$$

where *b* is the length of Burgers vector, *β* is the core cut-off parameter, *µ* is shear modulus, *υ* is the Poisson ratio, and *h* is the layer thickness parallel to the glide plane. Also, interfacial stress initiating due to elastic deformation in the interface area is stated by the gradient of the interfacial energy (γ) as a function of applied strain (ε) as *f* = γ + dγ/dε [86] also affects *σ_cls_*. The applied shear stress (*σ_cls_*) [17] incorporating interface stress is given as

(2) $$\sigma_{cls}=M\frac{\mu b}{8\pi h}\left(\frac{4-\vartheta }{1-\vartheta }\right)\left[\ln\frac{\beta h}{b}\right]-\frac{f}{h}$$

where *M* is the Taylor factor. The second term in Equation (2) is the interface stress (*f*/*h*), which typically increases from 20 MPa to 2 GPa with a decrease in *h* from 100 to 1 nm. This interface stress typically pre-stresses the individual layers in nanolaminates. This interfacial stress can complement or counteract the applied stress. For instance, in the case of applied tensile stress aligned along the interface, applied stress acts counter to interface stress, which is typically negative for metal systems [86]. In distinction, under applied compressive stress, the high interfacial stress (*f*/*h*) in the case of Cu(16)/Nb(16) ARB nanolaminate (low interfacial motion, especially along RD) will contribute to the plasticity of the nanolaminate. The compressive stress is what the Cu(16)/Nb(16) ARB nanolaminate mainly experiences during nanoindentation, which leads to high plasticity, as can be observed from the pile-up heights obtained during the nanoindentation experiments. Furthermore, the interfacial stress (*f*/*h*) is high in the case of RD compared to TD due to the crystallographic anisotropy along the RD and TD. Therefore, high interfacial stress (*f*/*h*) contributes more to plasticity and thus to higher pile-up along the RD compared to TD, as shown in Figure 9.

### 4.2. Pile-Up as a Measure of Anisotropy of Cu(16)/Nb(16) ARB Nanolaminate

**Figure 10:**
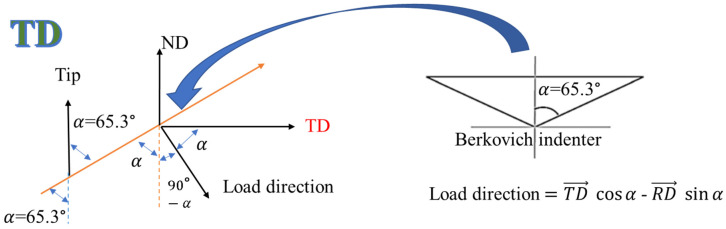
Direction of the load normal to the Berkovich indenter tip surface where the major axis of the indent was aligned along the TD, which was used to calculate the critical resolved shear stress (CRSS) (or Schmid factor) for the Cu layer (Table 3). Similar calculations were performed for RD. The angle (α) is the half-angle of 65.3° measured from the axis to one of the pyramid flats of the Berkovich tip.

Pile-up of material during indentation depends largely on the prevailing crystallographic texture of the nanolayer and on the lattice parameters at the interface. Moreover, the amount of pile-up also indicates a strong influence of the anisotropy as detected along the TD and RD in Cu(16)/Nb(16) ARB nanolaminate. For Cu(16)/Nb(16) ARB nanolaminate, the crystallographic axes aligned along the normal direction (ND) can vary from [1 1 2], [1 1 0] to [4 411] as a function of the copper layer thickness [42]. Likewise, for Niobium, the ND can vary from [1 1 2] to [3, 3, −8] as a function of the layer thickness. For the Cu(16)/Nb(16) ARB nanolaminate, the crystallographic axes along the ND, RD and TD were inferred from pole figures measured using a 4-axis diffractometer (see Figure 1 and Figure 2). For niobium, ND is [1 1 2], RD is [1 −1 0] and TD is [−1 −1 1] and for copper, ND is [1 1 2], RD is [−1 −1 1] and TD is [1 −1 0]. Here, the possible effect of crystallographic texture on pile-up could be obtained by documenting activated slip systems along TD and RD [87]. In particular, for FCC copper, there are 12 available slip planes. Typically, dislocation is introduced once the applied shear stress along the slip plane matches the CRSS. Therefore, the CRSS (or Schmid factor(*m*)) can be used to find the activated slip systems along the TD and RD (see Table 3 and Figure 10). The obtained Schmid factor is then normalized by ln(h_eff_/b)/h_eff_, where h_eff_ is the effective layer thickness and *b* is the Burger vector. In the case of Cu(16)/Nb(16) ARB nanolaminate, the CLS model is applied, where the stress (*σ*) is proportional to ~ln(h_eff_/b)/h_eff_. In the case of nanolaminate, if the slip system is perpendicular to the interface, the effective layer thickness will be the same as the actual layer thickness. However, when a slip system is inclined to the interface, the effective layer thickness will be different from the actual layer thickness. Therefore, the CLS stress (*σ*) could change with effective layer thickness despite having the same Schmid factor. Therefore, in order to consider the effect of effective layer thickness for slip systems, the Schmid factor is normalized by ln(h_eff_/b)/h_eff_ (see Table 3). Table 3 clearly shows that there were more activated slip systems along the RD compared to the TD for the Cu layer, as depicted by the non-zero values, which can be related to the high plasticity along the RD compared to the TD observed experimentally (see Figure 9). We are aware that the Schmid factor is related to uniaxial loading, which is not the case in our nanoindentation experiment, but we believe that such a calculation gives the general trend. Although the calculation of the critical resolved shear stress here takes into account the effect of the applied load on a single slip system and does not consider the interaction between the different slip systems or the complex stress field generated during the Berkovich indentation, the authors believe that the present analysis is reasonable to provide a preview and some manner of quantification of the high plasticity or pile-up extent that was indeed experimentally observed along the RD as compared to along the TD.

Table 4 shows the comparison between the experimental and simulated data, indicating a qualitative consistency. That is, the simulated pile-up heights (for different nanoindentation depths) along the TD are in general lower than those along the RD—in agreement with what we observed in the experiments. Here, a linear plasticity model was used for TD due to the higher yield strength (low plasticity), while power law plasticity was used for RD due to low yield strength (higher plasticity), which is in qualitative accord with the experimental data. The simulation results to a certain extent overestimated pile-up along the TD and underestimated it along the RD, which can be related to the different plasticity models used for the TD and RD of Cu(16)/Nb(16) ARB nanolaminate. Theoretically, the finite element method incorporating 3D crystal plasticity (CPFEM) should be applied to emulate Cu(16)/Nb(16) ARB nanolaminate plasticity and its associated anisotropy along the TD and RD to obtain more accurate results. Nevertheless, it could be very difficult to model non-uniformly activated slip systems due to the inhomogeneous stresses generated during the Berkovich indentation.

### 4.3. TEM + EDX Analysis of Cu(16)/Nb(16) ARB Nanolaminate

**Figure 11:**
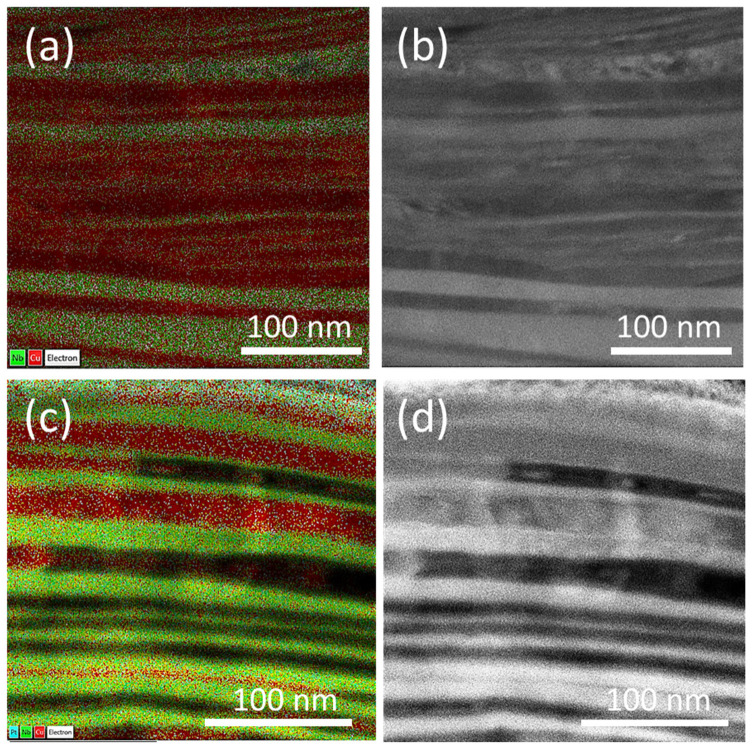
TEM + EDX image (red = Cu, green = Nb) of the representative Cu(16)/Nb(16) ARB nanolaminate (**a**,**b**), EDX and TEM for RD. TEM shows a possible fracture of the nanolayers (most likely Nb) due to the inhomogeneous deformation, and the other element (most likely Cu) starts filling the cavity in the broken nanolayers. This leads to the formation of layers into one large band (mostly red-colored in (**a**); about as thick as 5–8 typical individual nanolayers in this sample) as evident in the EDX image (**a**), instead of the layer-by-layer structure typically found in fabricated Cu(16)/Nb(16) ARB samples, or what is shown in images (**c**,**d**). (**c**,**d**) EDX and TEM for nanolayers representative of TD samples consisting mostly of layer-by-layer structures that are maintained due to more homogenous deformation.

TEM images with corresponding EDX maps of Cu(16)/Nb(16) ARB nanolaminates are shown in Figure 11. In the case of TD, the layers deform homogeneously (in a layer-by-layer manner, while layers just slide on each other on the interfaces to accommodate the loading), while in the case of RD, more disordered plastic deformation across the nanolayers is visible, especially in the Cu layers, as depicted in Figure 11a,b. This behavior can be attributed to the anisotropy of the Cu(16)/Nb(16) ARB nanolaminates. In general, during the fabrication of nanolaminates, similar lattice parameters are preferred for the epitaxial growth of alternating layers [71,88]. Any deviation in lattice parameters could cause elastic strain, which could introduce misfit dislocations to compensate for the built-up elastic strain, which is the case for incoherent Cu(16)/Nb(16) ARB interfaces [59,89,90,91]. In incoherent Cu(16)/Nb(16) ARB nanolaminates, the barrier to the motion of dislocations trapped in interfaces depends on the shear strength of the interface [92]. At low interfacial shear strength (along TD for Cu(16)/Nb(16) ARB nanolaminates), the sliding of interfaces generates positive force on dislocations, that allows core dispersion in interfaces to avoid the dislocation transfer across the interfaces, resulting in lower plasticity and more homogeneous deformation along the TD, as depicted in Figure 11c,d [93]. However, high shear strength of the interface along the RD will assist dislocation glide and climb at an interface, thus resulting in more plasticity and inhomogeneous deformation shown in Figure 11a,b. Thus, the high inhomogeneous and disordered plasticity depicted by RD is associated with the high pile-up compared to TD, as measured experimentally.

## 5. Conclusions

Nanoindentation was performed along the RD and TD of Cu(16)/Nb(16) ARB nanolaminates in this study, and the experimentally obtained pile-up data were compared with the 2D finite element modeling results as a function of indentation depth. It was found that the Cu(16)/Nb(16) ARB nanolaminate exhibited higher surface pile-up along the RD than along the TD, which was attributed to their anisotropy. The crystallographic anisotropy along the TD and the RD led to high yield strength and low overall plasticity along the TD compared to the RD. Moreover, the crystallographic anisotropy contributed to a high interfacial slip at the TD compared to the RD, which led to a lower co-deformation of the layers at the TD compared to the RD. These aspects led to higher accumulation along the RD compared to the TD of the Cu(16)/Nb(16) ARB nanolaminate. Our sample of 16 nm Cu/Nb ARB nanolaminate is one of the representative examples that followed confined layer slip (CLS) plasticity (100 < h < 10 nm), so the specific behavior of the hardness and pile up data of the 16 nm Cu/Nb ARB presented here should be similar to data for any layer thickness in the range of 100 < h < 10 nm. The authors do believe that a more detailed analysis using nanolaminates with layer thicknesses representative of all the three plasticity regimes should be performed and compared. The individual layer thickness of 16 nm was chosen in the present study as the overall aim of the present study was to investigate the plastic behaviour, and especially the interfacial sliding mechanisms of Cu/Nb ARB nanolaminates at smaller length scales (approaching the nanometre scale). The 16 nm Cu/Nb ARB samples had among the smallest individual layer thicknesses that were successfully produced with good morphology of the nanolayers and reasonable quality of the interfaces. Nevertheless, full investigations of the plastic behaviour and interfacial sliding mechanisms of the Cu/Nb ARB samples in the three plasticity regimes are indeed the path forward to the present study.

## Figures and Tables

**Table 1:** The properties of the Cu and Nb layers used in the nanoindentation simulation.

Material	Property	RD	TD
Cu	Young’s modulus, GPa	110	110
	Poisson’s ratio	0.34	0.34
	Yield strength, GPa	0.649	0.649
	Hardening rate, GPa	7.008	7.008
Nb	Young’s modulus, GPa	105	105
	Poisson’s ratio	0.4	0.4
	Yield strength, GPa	0.844	0.844
	Hardening rate, GPa	10.120	10.120

**Table 2:** The properties of the Cu(16)/Nb(16) ARB interface employed in the nanoindentation simulation.

Material	Property	RD	TD
Cu(16)/Nb(16) ARB Interface	Normal stiffness, mN/µm^2^	1 × 10^5^	1 × 10^5^
	Tangential stiffness, mN/µm^2^	1 × 10^5^	1 × 10^5^
	Normal strength, GPa	10	10
	Tangential strength, GPa	10	2 [56]
	Bond degradation type	linear	linear
	Bond degradation strain	0.3	0.3

**Table 3:** Schmid factor to analyze the activated slip systems along the TD and RD for the Cu layer.

Slip Directions	[1, −1, 0]	[−1, 0, 1]	[0, 1, −1]	[1, −1, 0]	[1, 0, 1]	[0, 1, 1]	[1, 1, 0]	[1, 0, −1]	[0, 1, 1]	[1, 1, 0]	[1, 0, 1]	[0, 1, −1]
Slip planes	(1, 1, 1)	(1, 1, 1)	(1, 1, 1)	(1, 1, −1)	(1, 1, −1)	(1, 1, −1)	(1, −1, 1)	(1, −1, 1)	(1, −1, 1)	(−1, 1, 1)	(−1, 1, 1)	(−1, 1, 1)
m(RD) (nm)	0	0.198	−0.198	0	0.984	0.984	1.17	−0.031	1.207	1.175	1.207	−0.031
m(TD) (nm)	−2.144	3.399	−1.255	0	0	0	0.582	−0.425	1.008	1.577	2.003	−0.425

**Table 4:** Comparison of experimental and simulation pile-up data.

Direction	Depth (nm)	Experimentally Obtained Pile-Up (nm)	Simulated Pile-Up (nm)
TD	200	10.86	16
TD	300	15.88	24
TD	400	28.28	28
RD	200	15.45	19
RD	300	39.44	28
RD	400	49.77	37

## Data Availability

The data presented in this study are available upon request from the corresponding author.

## Supplementary Materials

The following are available online at https://www.mdpi.com/article/10.3390/nano12030308/s1, Figure S1: Pole Figure measurement using 4 axis diffractometer, Figure S2: Graphs depict (a) Hardness and (b) reduced elastic modulus for both TD and RD with respect to indentation depths, Figure S3: Pileup distribution along (a) RD and (b) TD for the indenter depth of 200 nm. The unit of the data shown in legend is GPa, Figure S4: Pileup distribution along (a) RD and (b) TD for the indenter depth of 300 nm. The unit of the data shown in legend is GPa, Video S1: RD200nm.mp4, Video S2: RD300nm.mp4, Video S3: RD400nm.mp4, Video S4: TD200nm.mp4, Video S5: TD300nm.mp4, Video S6: TD360nm.mp4.
