# Supplementary material for: Crystallographic Anisotropy Dependence of Interfacial Sliding Phenomenon in a Cu(16)/Nb(16) ARB (Accumulated Rolling Bonding) Nanolaminate
Source: Nanomaterials (Basel). 2022 Jan 18;12(3):308. doi: 10.3390/nano12030308 (PMC8840588; doi:10.3390/nano12030308)
Supplement: Supplementary file 1 [file nanomaterials-12-00308-s001.zip › Supplementary data_18_1_22.pdf]

Supplementary data

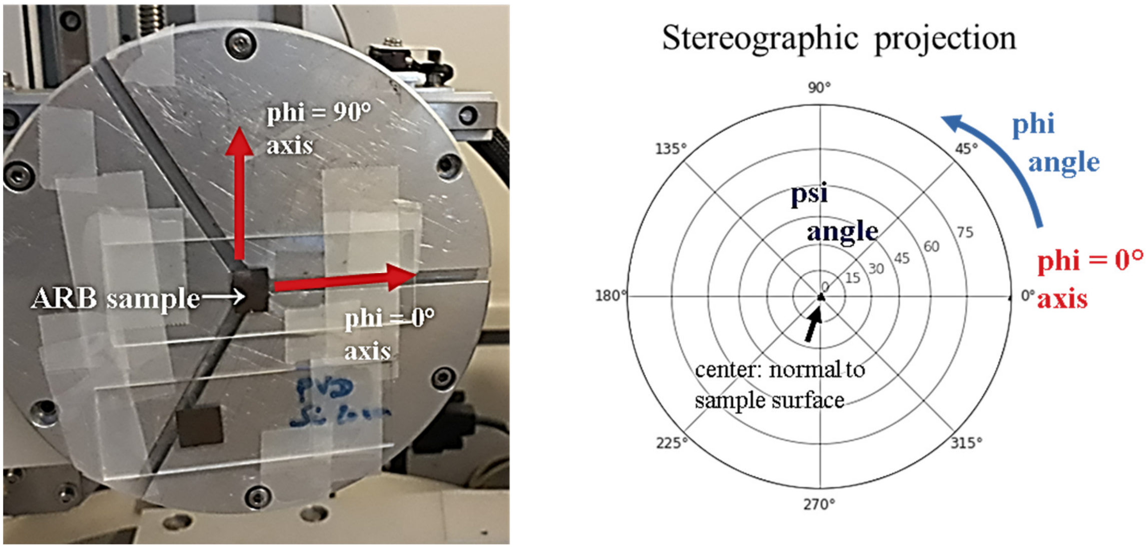

Figure S1. Pole Figure measurement using 4 axis diffractometer.

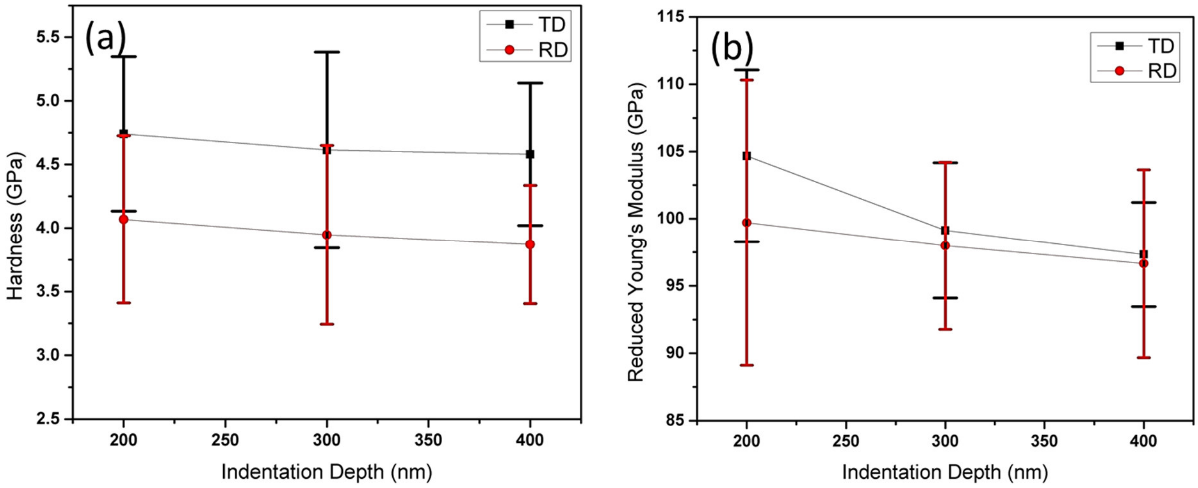

Figure S2. Graphs depict (a) Hardness and (b) reduced elastic modulus for both TD and RD with respect to indentation depths.

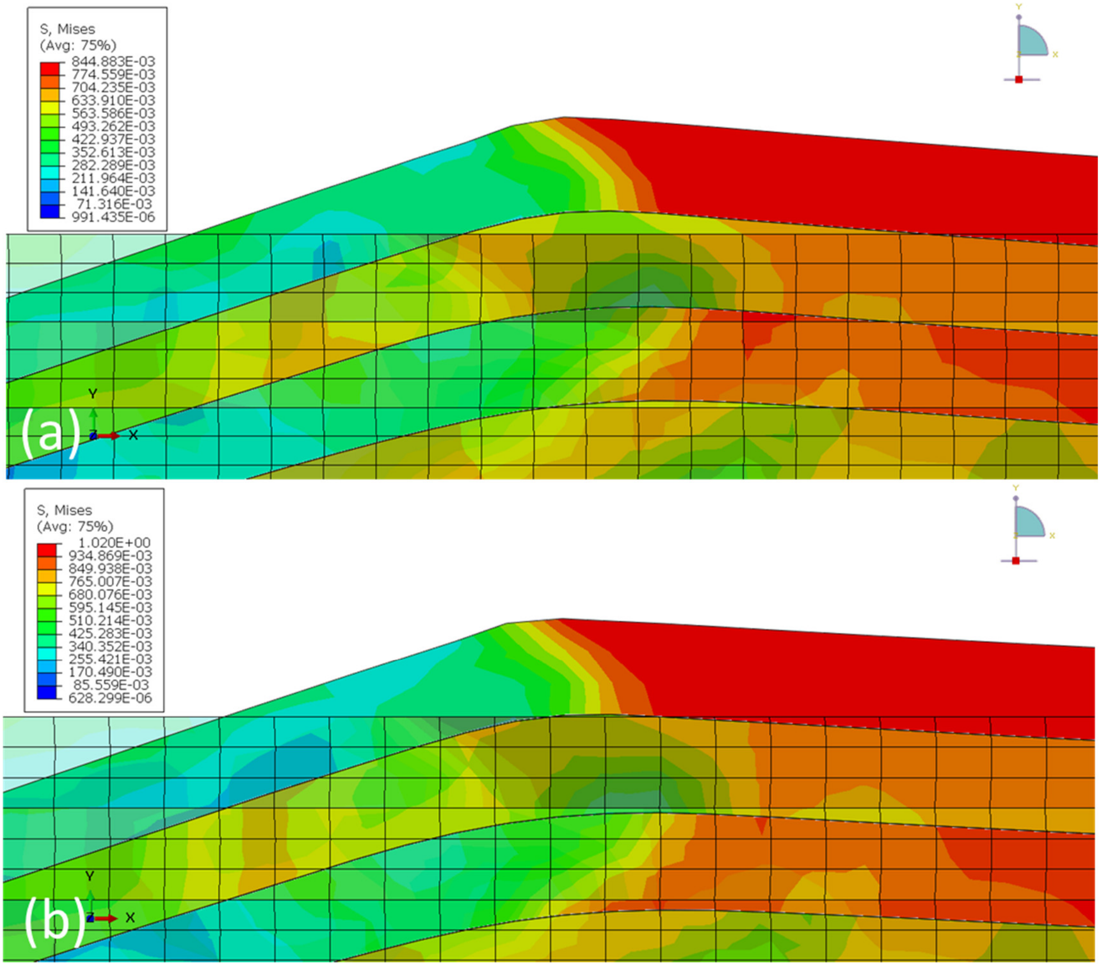

Figure S3. Pileup distribution along (a) RD and (b) TD for the indenter depth of 200 nm. The unit of the data shown in legend is GPa.

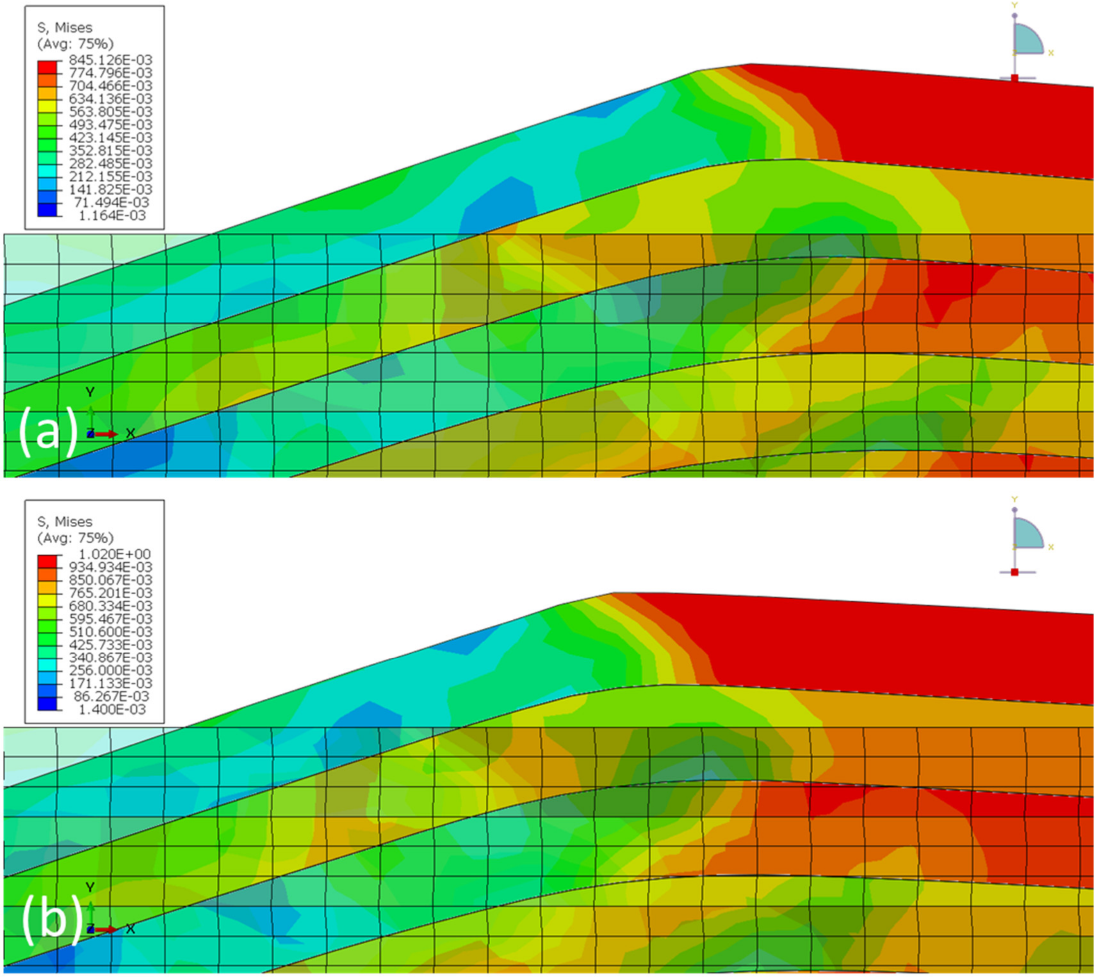

Figure S4. Pileup distribution along (a) RD and (b) TD for the indenter depth of 300 nm. The unit of the data shown in legend is GPa.
